# Oral health and oral health-related quality of life in community-dwelling older adults: associations with geriatric syndromes in a retrospective study

**DOI:** 10.1007/s00784-026-06842-7

**Published:** 2026-03-28

**Authors:** Marc Auerbacher, Madeleine Carina Beck, Sabine Schluessel, Karin Christine Huth, Falk Schwendicke, Vinay Pitchika, Michael Drey

**Affiliations:** 1 Department of Conservative Dentistry, Periodontology and Digital Dentistry, University Hospital, LMU Munich, Goethestraße 70, 80336 Munich, Germany; 2 Department of Medicine IV, Geriatrics, University Hospital, LMU Munich, Ziemssenstraße 5, 80336 Munich, Germany

**Keywords:** Geriatric syndromes, Older adults, Oral health, Oral health-related quality of life

## Abstract

**Objective:**

This study characterises the oral and general health of community-dwelling older adults and examines the relationship between geriatric syndromes, oral health, and oral health-related quality of life (OHRQoL).

**Materials and methods:**

The study included 138 patients (mean age 81 years, 73% women) from the MUnich SArcopenia Registry (MUSAR) database, divided into three age groups (< 80, 80–85, > 85 years). Oral health was assessed, for example, with decayed, missing, and filled teeth (DMFT), the Periodontal Screening Index (PSI), and the Plaque Index (PI). Geriatric parameters included the Charlson Comorbidity Index (CCI), probable sarcopenia, frailty, and the Mini Nutritional Assessment (MNA). OHRQoL was evaluated using two patient-reported outcome measures (PROMs). Alongside statistical tests comparing age groups, linear regression analyses were performed to examine associations between various parameters.

**Results:**

Oral health was generally at a moderate level with a mean DMFT of 19.3 ± 6.4, with hardly any variation across age groups. OHRQoL was significantly influenced, among others, by Activities of Daily Living (ADL) (β = -0.202, 95%CI -0.313 to -0.092) and MNA (β = -0.594, 95%CI -1.054 to -0.133), whereas frailty was strongly associated with dental health measured by DMFT Index (β = 4.118, 95%CI 1.445 to 6.790).

**Conclusions:**

We confirm associations between general and oral health in older individuals.

**Clinical Relevance:**

Links between oral and general health in community-dwelling older adults highlight the need for interdisciplinary collaboration. Recognising the factors affecting both health and quality of life enables comprehensive care and may be highly beneficial for older patients.

## Introduction

Oral health is a key determinant of general health and well-being throughout all stages of life [1]. With advancing age, maintaining functions such as speaking, eating, and smiling as long as possible is essential to preserve full participation in social life and an adequate quality of life (QoL) [2].

Despite the growing recognition of the links between oral health, overall health, and well-being, the interdisciplinary overlap between geriatric and dental care settings receives only limited attention in both research and practice [3]. Furthermore, extant literature tends to focus on collecting data from institutionalized patients, as data on very old community-dwellers appears to be more sporadic.

Numerous studies indicate that physical and oral frailty are interdependent and may exacerbate each other [4]. The impact of oral health on physical factors related to geriatric syndromes, such as weight and muscle loss, disability and mobility, systemic disease, malnutrition, psychological factors and cognition, QoL, and even effects on mortality was demonstrated [5]. The mutual influence and interaction between oral health and geriatric parameters are important factors when evaluating current health status and the further development of geriatric syndromes [6].

Nevertheless, the relationship between geriatric syndromes and oral health is not yet fully understood [3]. This interaction, however, seems to be bidirectional. Age-related diseases can not only have direct negative effects on oral health, but also frequently lead to a reduced or completely lost ability to perform oral hygiene independently due to motor, sensory, and/or cognitive impairments [7]. Increased attention to oral health, alongside appropriate therapeutic interventions, could improve clinical outcomes for older adults [3].

With age, the risk of geriatric syndromes rises [8, 9], often leading to greater care needs and loss of independence [10]. Individuals > 80 years constitute the largest share of care level 3, and most of the residents in long-term care facilities [11]. Therefore, community-dwelling older adults should receive special attention, as they are accessible to medical care, and both preventive and interventional measures are feasible. Early identification of functional capacities and impairments through multidimensional, interdisciplinary geriatric assessments may reduce dependency risk by enabling tailored interventions [12].

A range of validated clinical and diagnostic parameters is available for the comprehensive assessment of oral health in older adults [13]. Despite their advanced age, examinations were feasible because the cohort showed no or only mild cognitive impairments, enabling reliable data collection and valuable insights into this age group. Various studies have demonstrated the importance of adopting a holistic approach and combining the results of both dental and geriatric assessments for causal research and therapy. Those studies found an association between bad oral health conditions and adverse long-term systemic health outcomes [14].

Early recognition and responsiveness to this issue may reduce long-term suffering and alleviate the burden on the healthcare system. As the population ages in most countries and the prevalence of multiple chronic conditions increases, complex challenges arise for social and healthcare services as well as long-term care facilities [15]. Providing adequate care for community-dwelling older adults and preserving this status is of particular relevance. Furthermore, it remains essential to ensure that the older population can achieve the highest possible QoL, which necessitates an awareness of the factors influencing it [16]. Insights from the older cohort can directly benefit the older adults and offer advantages for younger populations by revealing long-term consequences and enabling early interventions [17].

The MUnich SArcopenia Registry (MUSAR) collected sociodemographic, anthropometric, functional, and laboratory data from patients at the geriatric clinic, together with a dental assessment, both conducted at the Ludwig Maximilian University (LMU) Munich [18].

As comprehensive epidemiological data on oral health in this age group in Germany were last collected in 2014 [19], we aimed to present the oral health status of our cohort in detail using standardized, objective parameters. In addition, we described the general health status of the participants, with a focus on typical geriatric diseases. Patient-reported outcome measures (PROMs) were used to assess oral health-related quality of life (OHRQoL) and self-rated health status. We also examined age-related trends within the cohort. A further objective was to explore interactions between geriatric diseases, oral health, and OHRQoL. We hypothesize that within the older cohort, poorer oral health parameters are associated with adverse geriatric conditions, and that this interplay significantly reduces OHRQoL. Such insights may foster mutual understanding, facilitate early risk identification, and support timely interdisciplinary interventions to maintain overall health and well-being.

## Materials and methods

### Participants

The study included 138 patients (73% female; mean age: 81 ± 5.5 years, range 66–97 years) at the geriatric day care-hospital at the LMU hospital in Munich, Germany. MUSAR was used to source patient data from February 2021 to February 2025. No formal sample size calculation was performed; a comprehensive sample of all eligible participants during the study period was included. Inclusion criteria were participation in a comprehensive geriatric assessment of dental and oral health and the ability to provide informed consent. Exclusion criteria were: no participation in the geriatric assessment of dental and oral health, and inability to give informed consent. Data analysis and registry inclusion were approved by the Ethics Committee of the Medical Faculty of LMU Munich, Germany (Ethics votes No. 25–0669 and No. 17–874).

### Oral health examination

As part of the geriatric assessment of dental and oral health, clinical dental students conducted oral health examinations under the strict supervision of experienced clinicians, following detailed manuals to ensure a standardised assessment. The following clinical indices were collected:

the number of decayed, missing, and filled teeth (DMFT), periodontal status (Periodontal Screening Index, PSI), oral hygiene (Plaque Index, Silness and Loe, 1964), presence of removable dental protheses, maximal bite force, measured with a prototype of the Bredent medical company [20], and temporomandibular dysfunction (TMD) using the screening protocol of the German Association for Functional Diagnostics and Therapy [21]. Furthermore, the Oral Health Assessment Tool (OHAT) was used to evaluate general oral health [22].

### Geriatric assessment

The general health condition was routinely evaluated and recorded by trained medical staff as part of the patient’s stay at the geriatric day hospital. Data collection included the: the Charlson Comorbidity Index (CCI) for measuring comorbidity, an evaluation of the ability to perform activities of daily living (ADLs) using the Barthel Index (BI), the Mini Nutritional Assessment (MNA) to assess the nutritional status, cognitive function evaluated with the Mini Mental State Examination (MMSE), and a depression screening using the Beck Depression Inventory (BDI-II).

The Fried Phenotype (Original Fried) was used to classify frailty, taking into account unintentional weight loss, self-reported exhaustion, weakness (grip strength), slow walking speed, and low physical activity [23]. Probable sarcopenia was diagnosed following the 2019 definition of the European Working Group on Sarcopenia in Older People (EWGSOP2), involving reduced muscle strength measured using the chair rise test and hand grip strength test [24]. Dual-energy X-ray absorptiometry (DXA) was used to determine the lowest bone mineral density with t-score from the lumbar spine or femur for diagnosing osteoporosis according to World Health Organization criteria [25].

### OHRQoL and self-rated health

Questionnaire-based PROMs were used to assess OHRQoL using the Oral Health Impact Profile (OHIP) and the Geriatric Oral Health Assessment Index (GOHAI) [26]. The OHIP is a validated instrument measuring the subjective burden of oral health problems across multiple dimensions, with a total score ≥ 11 indication “poor OHRQoL” [26, 27], while the GOHAI, developed for older adults and focusing on functional and psychological aspects, uses a score ≤ 50 to define “poor OHRQoL” [28, 29]. Furthermore, the EuroQol EQ5D visual analogue scale (EQ5D-VAS) allowed patients to rate their current health status subjectively on a printed scale ranging from 0 to 100 [30].

### Other items

Additional recorded parameters included sex, year of birth, marital status, alcohol consumption, smoking, body mass index (BMI), the number of medications taken, and the frequency of dentist visits. Education level, in line with the German school system, was recorded as lower secondary (Hauptschule), upper secondary (Realschule/ISCED 2), tertiary entrance qualification (Abitur/ISCED 3), and no formal school qualification.

### Statistical analysis

The dataset was anonymised before analysis. Age was calculated using the year of birth and the date of the dental examination. The cohort was divided into age groups: < 80 years (*n* = 49), 80–85 years (*n* = 61), and > 85 years (*n* = 28), to examine heterogeneity within the older cohort. Quantitative variables were presented using the mean and standard deviation (mean ± SD) and categorical variables as frequencies (n, %). Variables were tested for normality using the Shapiro–Wilk test. Significant differences between the age groups for quantitative variables were calculated using ANOVA, for non-normally distributed variables, the Kruskal–Wallis test, and for categorical variables, the chi-squared test. Furthermore, linear regression models were used to examine associations between geriatric syndromes and oral health status. To assess multicollinearity, variance inflation factors (VIFs) and pairwise Spearman correlation analyses were calculated for predictors included in each outcome-specific regression model (DMFT and OHIP). Homoscedasticity of residuals was assessed and confirmed. False discovery rate (FDR) correction was applied. The residuals of each regression model were assessed for normality. A post hoc power analysis was conducted for the multiple linear regression models with 15 predictors, an alpha level of 0.05, and an effect size of f² = 0.15. For the DMFT model (*n* = 111), the achieved power was 66.5%, whereas for the OHIP model (*n* = 79), the achieved power was 35.9%. Given the limited power for the OHIP model, these analyses should be considered exploratory. All statistical analyses were performed using SPSS 30.0 software (IBM Corp., Armonk, NY, USA), with a significance level set at *p* < 0.05. Missing data were handled using a complete case analysis approach. No imputation was performed. No additional sensitivity analyses were conducted, as the study was designed as a full sample. However, regression models were adjusted for relevant parameters to account for potential confounding.

### Bias

Potential sources of bias include selection bias resulting from recruitment at a single center, with geriatric day hospital attendees likely representing a relatively health-conscious and functionally preserved subset and dental service uptake potentially further limiting generalizability to frailer populations. Information bias from self-reported measures, and residual confounding from unmeasured variables should also be considered. Missing data were handled using complete-case analysis. Although this approach can introduce bias if data are not missing completely at random, most variables had less than 7% missing values, with only bone mineral density (13%) and BDI (11.6%) exceeding this. Given these low proportions, any resulting bias is likely minimal.

This study is reported in accordance with the Strengthening the Reporting of Observational Studies in Epidemiology (STROBE) guidelines.

## Results

### Participant characteristics

Table 1 summarizes the baseline characteristics of the 138 participants, with the majority being female and an average age of 81 years. Most of the individuals were either widowed or married. 37.4% reported regular alcohol consumption, only 2.3% were smokers. The mean BMI was 26.9 ± 5.4.

**Table 1:** Baseline characteristics of participants

Variable	All (*N* = 138)	< 80 years (*n* = 49)	80–85 years (*n* = 61)	> 85 years (*n* = 28)	*P*-value
Demographics					
Age, mean ± SD	81.0 ± 5.5	75.4 ± 2.8	82.0 ± 1.4	88.8 ± 3.5	**< 0.001** ^**c**^
Women, *n* (%)	101 (73.2)	35 (71.4)	41 (67.2)	25 (89.3)	.087^b^
Education, *n* (%)					.252^b^
Lower secondary	47 (36.7)	14 (29.8)	27 (48.2)	6 (24)	
Upper secondary	40 (31.3)	16 (34)	15 (26.8)	9 (36)	
Tertiary entrance	39 (30.5)	17 (36.2)	13 (23.2)	9 (36)	
No formal school qualification	2 (1.6)	0	1 (1.8)	1 (4)	
Marital status, *n* (%)					**< 0.001** ^**b**^
Single	18 (13.4)	11 (22.4)	5 (8.6)	2 (7.4)	
Married	45 (33.6)	17 (34.7)	23 (39.7)	5 (18.5)	
Permanent partnership	2 (1.5)	2 (4.1)	0	0	
Separated	19 (14.2)	11 (22.4)	6 (10.3)	2 (7.4)	
Widowed	50 (37.3)	8 (16.3)	24 (41.4)	18 (66.7)	
Alcohol consumption, *n* (%)	49 (37.4)	16 (33.3)	23 (40.4)	10 (38.5)	.754^b^
Smoking, *n* (%)	3 (2.3)	2 (4.2)	1 (1.8)	0	.479^b^
BMI, mean ± SD	26.9 ± 5.4	27.5 ± 6.2	26.4 ± 5.1	26.6 ± 4.4	.589^a^

All: all participants; BMI: Body Mass Index

^a^ One-way ANOVA

^b^ Chi-square test

^c^ Kruskal-Wallis test

Bold *p*-values are significant at 5%

Complete case analysis; no imputation

### Health status

As shown in Table 2, participants had a mean DMFT Score of 19.3 ± 6.4, increasing slightly across age groups. According to the OHAT, 48.6% of individuals exhibited poor oral health (score ≥ 3). The majority (55.2%) had a PSI of ≥ 3, indicating suspected periodontitis. No significant differences were observed across age groups in terms of oral hygiene measured by Plaque Index. Maximal bite force was significantly lower in the oldest than in the other two groups (*p* = 0.018). 42.3% were removable dental protheses users, while 18 participants had a positive TMD screening result. Most reported visiting a dentist at least once a year. Poor OHRQoL was observed in 12.3% of participants based on OHIP scores and in 22.5% when measured using the GOHAI. Spearman correlation analysis revealed a significant negative association between OHIP and GOHAI scores (*r* = − 0.56, *p* < 0.001), consistent with the inverse scaling of the two instruments and indicating that they capture overlapping but distinct domains of oral health–related quality of life. Table 2 presents that the mean number of medications among all participants was 9.5 ± 3.7. According to the CCI, the mean comorbidity count was 2 ± 2. 69.6% of participants met probable sarcopenia criteria. Pre-frailty/frailty was identified in 76.1% of individuals. Bone mineral density showed a mean t-score of -1.75 ± 1.39. Overall, participants demonstrated functional independence (ADL; 92 ± 10), good nutritional status (MNA; 11 ± 2.7), preserved cognitive function (MMSE; 27.4 ± 2.6), and an absence of depressive symptoms (BDI; 12.1 ± 7.6). There were no substantial differences in clinical health status among age groups. Similarly, the EQ5D-VAS (59 ± 19) remained relatively stable across age groups.

**Table 2:** Oral and clinical parameters

Variable	All (*N* = 138)	< 80 years (*n* = 49)	80–85 years (*n* = 61)	> 85 years (*n* = 28)	*P*-value
Oral Health					
DMFT, mean ± SD	19.3 ± 6.4	18.5 ± 6.4	19.7 ± 6.6	20.1 ± 6.2	.309^c^
OHAT, mean ± SD	2.67 ± 1.96	2.76 ± 2.31	2.74 ± 1.83	2.39 ± 1.57	.700^a^
Poor oral health, *n* (%)	67 (48.6)	24 (49)	30 (49.2)	13 (46.4)	.969^b^
Suspected periodontitis, *n* (%)	74 (55.2)	20 (43.5)	39 (65)	15 (53.6)	.086^b^
Plaque Index, mean ± SD	1.88 ± 0.96	1.98 ± 1.01	1.83 ± 0.97	1.85 ± 0.88	.622^c^
Positive TMD Screening, *n* (%)	18 (13.5)	8 (16.7)	9 (15.5)	1 (3.7)	.243^b^
Removable dental protheses, *n* (%)	58 (42.3)	20 (41.7)	24 (39.3)	14 (50)	.636^b^
Max. bite force^d^ (*N*), mean ± SD	596 ± 516	653 ± 538	629 ± 550	420 ± 350	**0.018** ^**c**^
Visited dentist ≥ 1×/year, *n* (%)	116 (84.7)	37 (77.1)	55 (90.2)	24 (85.7)	.168^b^
Oral Health-related Quality of Life (OHRQoL)					
OHIP, mean ± SD	4.5 ± 6.5	5.4 ± 6.9	4.6 ± 6.6	2.9 ± 5.2	.161^c^
Poor OHRQoL, *n* (%)	17 (12.3)	8 (16.3)	6 (9.8)	3 (10.7)	.565^b^
GOHAI, mean ± SD	53 ± 8	52 ± 9	53 ± 9	55 ± 5	.460^c^
Poor OHRQoL, *n* (%)	31 (22.5)	15 (30.6)	14 (23)	2 (7.1)	.059^b^
Clinical Health Status					
Number of medications, mean ± SD	9.5 ± 3.7	9.3 ± 3.8	9.8 ± 3.6	9.0 ± 3.8	.548^a^
CCI, mean ± SD	2 ± 2	2 ± 2	2 ± 2	2 ± 1	.814^c^
Probable Sarcopenia, *n* (%)	96 (69.6)	29 (59.2)	46 (75.4)	21 (75.0)	.144^b^
Pre-Frailty, *n* (%)	105 (76.1)	33 (67.3)	49 (80.3)	23 (82.1)	.199^b^
Bone mineral density, mean ± SD	-1.75 ± 1.39	-1.97 ± 1.26	-1.60 ± 1.59	-1.69 ± 1.10	.378^c^
ADL, mean ± SD	92 ± 10	93 ± 7.58	92 ± 11	90 ± 10	.512^c^
MNA, mean ± SD	11.0 ± 2.7	11.0 ± 2.9	10.7 ± 2.7	11.6 ± 2.4	.351^c^
MMSE, mean ± SD	27.4 ± 2.6	27.9 ± 2.7	27.1 ± 2.3	27.2 ± 2.6	**0.037** ^**c**^
BDI, mean ± SD	12.1 ± 7.6	13.1 ± 8.4	11.6 ± 6.9	11.4 ± 8.0	.628^c^
Self-rated Health					
EQ5D-VAS, mean ± SD	58 ± 19	64 ± 20	55 ± 19	57 ± 14	.150^c^

All: all participants; DMFT: Decayed Missing Filled Teeth; OHAT: Oral Health Assessment Tool (≥ 3 “poor oral health”), Suspected periodontitis: Periodontal Screening Index ≥ 3; TMD: Temporomandibular Dysfunction; Removable dental protheses: partial and complete dental protheses, OHIP: Oral Health Impact Profile (≥11 “poor OHRQoL”); GOHAI: Geriatric Oral Health Assessment Index (≤ 50 “poor OHRQoL”); CCI: Charlson Comorbidity Index; Probable Sarcopenia: 2019 EWGSOP2 criteria (handgrip strength < 27 kg for men or < 16 kg for women, or chair rise test ≥ 15 s); Pre-Frailty: Fried-Phenotype (≥1 criterion positive); Bone mineral density: T-Score; ADL: Activities of Daily Living (Barthel); MNA: Mini Nutritional Assessment; MMSE: Mini-Mental-State Examination; BDI: Beck Depression Inventory; EQ5D-VAS: EuroQoL 5 Dimension Visual Analogue Scale

^a^ One-way ANOVA

^b^ Chi-square test

^c^ Kruskal-Wallis-Test

^d^ *n* = 87

Bold *p*-values are significant at 5%

Complete case analysis; no imputation

### Determinants of oral health and OHRQoL

Multiple linear regression analysis were performed to estimate the determinants of DMFT and OHIP. As shown in Table 3, pre-frail/frail status was significantly associated with an increased DMFT (β = 4.118, 95%CI 1.445 to 6.790). In Table 4, lower OHRQoL was significantly associated with greater functional dependence (ADL; β = -0.202, 95%CI -0.313 to -0.092), impaired nutritional status (MNA; β = -0.594, 95%CI -1.054 to -0.133), and depressive symptoms (BDI; β = 0.282, 95%CI 0.128 to 0.435). Furthermore, lower self-rated health was associated with reduced OHRQoL (EQ5D-VAS; β = -0.113, 95%CI -0.174 to -0.053). These significant associations remained robust across all models.

Adjusted R² values for the fully adjusted models (Model 3) were generally small to moderate, reflecting the multifactorial nature of oral health outcomes. For DMFT, values ranged from − 0.03 to 0.05, and for OHIP from − 0.03 to 0.14, indicating that the models explained a modest proportion of variance in dental health and OHRQoL.

**Table 3:** Linear regression analysis with DMFT as the dependent variable

		Model 1	Model 2	Model 3
Independent variable	*n*	β (95% CI)	β (95% CI)	β (95% CI)
Clinical parameters				
Number of medications	126	0.395 (0.101–0.688)	0.398 (0.099–0.697)	0.407 (0.101–0.713)
CCI	123	0.668 (0.120–1.216)	0.721 (0.141–1.301)	0.768 (0.177–1.359)
Probable Sarcopenia	126	1.739 (-0.744–4.221)	1.587 (-0.918–4.092)	1.805 (-0.853–4.464)
Pre-Frailty	126	**4.175 (1.584–6.766)**	**4.080 (1.429–6.730)**	**4.118 (1.445–6.790)**
Bone mineral density	111	-0.745 (-1.559–0.069)	-0.790 (-1.615–0.035)	-0.886 (-1.763 – -0.008)
ADL	126	-0.114 (-0.226 – -0.001)	-0.105 (-0.219–0.010)	-0.116 (-0.235–0.002)
MNA	125	-0.011 (-0.442–0.421)	-0.010 (-0.442–0.422)	0.001 (-0.485–0.487)
MMSE	126	-0.342 (-0.780–0.096)	-0.311 (-0.756–0.133)	-0.316 (-0.779–0.146)
BDI	114	0.031 (-0.131–0.192)	0.043 (-0.122–0.207)	0.059 (-0.109–0.227)
EQ5D-VAS	121	-0.079 (-0.140 – -0.018)	-0.078 (-0.141 – -0.015)	-0.079 (-0.142 – -0.016)

Model 1: unadjusted; Model 2: adjusted for sex and age; Model 3: adjusted for sex, age, BMI, education, and smoking

Bootstrap 95% CI (confidence intervals) were estimated using 1000 resamples

CCI: Charlson Comorbidity Index; Probable Sarcopenia: 1 = 2019 EWGSOP2 criteria (handgrip strength < 27 kg for men or < 16 kg for women, or the chair rise test ≥ 15 s), 0 = criteria not met; Pre-Frailty: 1 = Fried-Phenotype (≥ 1 criterion positive), 0 = criteria not met; Bone mineral density: T-Score; ADL: Activities of Daily Living (Barthel); MNA: Mini Nutritional Assessment; MMSE: Mini-Mental-State Examination; BDI: Beck Depression Inventory; EQ5D-VAS: EuroQoL 5 Dimension Visual Analogue Scale

Bold *p*-values are significant at 5%. FDR correction according to Benjamini–Hochberg was applied

Complete case analysis; no imputation

Model assumptions were checked, including residual normality, homoscedasticity, and absence of multicollinearity (VIF)

**Table 4:** Linear regression analysis with OHIP as the dependent variable

		Model 1	Model 2	Model 3
Independent variable	*n*	β (95% CI)	β (95% CI)	β (95% CI)
Oral parameters				
DMFT	126	0.067 (-0.110–0.243)	0.072 (-0.103–0.247)	0.074 (-0.104–0.252)
Suspected Periodontitis	124	1.211 (-1.111–3.533)	0.990 (-1.298–3.277)	1.102 (-1.263–3.467)
Plaque Index	122	0.714 (-0.444–1.872)	0.484 (-0.682–1.651)	0.458 (-0.740–1.656)
Removable dental protheses	127	2.385 (0.132–4.638)	2.092 (-0.150–4.334)	2.249 (-0.050–4.549)
Max. bite force	79	-0.002 (-0.006–0.001)	-0.003 (-0.006–0.000)	-0.003 (-0.006–0.000)
Visited dentist ≥ 1×/year	127	-2.001 (-5.031–1.029)	-1.366 ( -4.399–1.667)	-1.181 (-4.287–1.924)
Clinical parameters				
Number of medications	128	0.351 (0.061–0.642)	0.305 (0.014–0.596)	0.333 (0.034–0.633)
CCI	125	0.637 (0.095–1.179)	0.469 (-0.103–1.041)	0.478 (-0.111–1.067)
Probable Sarcopenia	128	-0.818 (-3.277–1.641)	-0.806 (-3.241–1.628)	-0.923 (-3.523–1.678)
Pre-Frailty	128	2.523 (-0.096–5.143)	3.099 (0.496–5.702)	3.165 (0.528–5.801)
Bone mineral density	113	-0.055 (-0.767–0.657)	-0.103 (-0.826–0.619)	0.044 (-0.729–0.817)
ADL	128	**-0.183 (-0.290 – -0.075)**	**-0.188 (-0.295 – -0.081)**	**-0.202 (-0.313 – -0.092)**
MNA	127	**-0.550 (-0.963 – -0.138)**	**-0.528 (-0.934 – -0.121)**	**-0.594 (-1.054 – -0.133)**
MMSE	128	0.106 (-0.330–0.542)	0.132 (-0.302–0.566)	0.172 (-0.281–0.625)
BDI	116	**0.304 (0.153–0.456)**	**0.277 (0.126–0.427)**	**0.282 (0.128–0.435)**
EQ5D-VAS	123	**-0.115 (-0.175 – -0.055)**	**-0.112 (-0.172 – -0.051)**	**-0.113 (-0.174 – -0.053)**

Model 1: unadjusted; Model 2: adjusted for sex and age. Model 3: adjusted for sex, age, BMI, education, and smoking

Bootstrap 95% CI (confidence intervals) were estimated using 1000 resamples

DMFT: Decayed Missing Filled Teeth; Suspected Periodontitis: 1 = Periodontal Screening Index ≥3, 0 = Periodontal Screening Index < 3; Removable dental protheses: 1 = partial and complete dental protheses, 0 = no dental protheses; CCI: Charlson Comorbidity Index; Probable Sarcopenia: 1 = 2019 EWGSOP2 criteria (handgrip strength < 27 kg for men or < 16 kg for women, or the chair rise test ≥ 15 s), 0 = criteria not met; Pre-Frailty: 1 = Fried-Phenotype (≥ 1 criterion positive), 0 = criteria not met; Bone mineral density: T-Score; ADL: Activities of Daily Living (Barthel); MNA: Mini Nutritional Assessment; MMSE: Mini-Mental-State Examination; BDI: Beck Depression Inventory; EQ5D-VAS: EuroQoL 5 Dimension Visual Analogue Scale

Bold *p*-values are significant at 5%. FDR correction according to Benjamini–Hochberg was applied

Complete case analysis; no imputation

Model assumptions were checked, including residual normality, homoscedasticity, and absence of multicollinearity (VIF)

## Discussion

### Oral health, OHRQoL, and geriatric syndromes: interrelations

Our research revealed poor oral health, suspected periodontitis, and tooth loss with a need for removable dental protheses in about half of the participants. Although our cohort exhibited slightly better mean oral health status than a comparable age group in the fifth German Oral Health Study (DMS V, 2014), a nationwide cross-sectional study [19], it appears to be representative of this age group. Within the studied age groups, oral health and hygiene do not appear to decline further with increasing age. Conversely, other studies have reported an association between age and impaired oral function [31]. As we did not observe such an association, this relationship may be driven by age-related comorbidities rather than age itself. Oral health was associated with overall health and functional status, both of which often decline in later life [23].

Whereas the clinical parameters revealed a high proportion of probable sarcopenia and pre-frailty, the functional, cognitive, mental, and nutritional status were generally well preserved. Participants of all age groups perceived their general health status on EQ5D-VAS as moderate.

In line with our results, research has shown that a pre-frail/frail status significantly shows a direct association with poorer oral health [32]. This further indicates an association between oral health and the patient’s general physical condition than with age.

Declined oral health can be associated with compromise chewing ability, which may result in insufficient intake of key nutrients, including proteins and vitamins, thereby heightening the risk of frailty [33, 34]. Moreover, elevated systemic inflammatory markers, such as IL-6 and CRP, could be associated with muscle loss and reduced physical activity and could thereby be linked to frailty [35].

Depending on the assessment instrument applied, over 20% of participants were classified as having poor OHRQoL. Despite the fact that this proportion is lower than that reported in studies of older adults from other countries, general well-being and QoL cannot be separated, and efforts should therefore aim to reduce this number further [36].

Although the cohort performed well in these measures, variations in ADL, MNA, and BDI scores were found to impact OHRQoL significantly. Expressed per clinically meaningful unit change a 10-point increase in ADL score and a 5-point increase in MNA score were associated with approximately 2- and 3-point lower OHIP scores, respectively, whereas a 5-point increase in BDI score was associated with approximately 1.4-point higher OHIP scores. That indicates that greater limitations in functional status, nutrition, and mental health tend to coincide with greater perceived impairments in oral health. The correlation between OHIP and MNA identified among community-dwellers was also shown in previous studies in nursing home residents [37]. While our analysis did not consider the individual OHIP domains, the aforementioned study reports “discomfort while eating” as the most significant contributor to a decreased MNA score [37]. Similar associations between OHRQoL and depressive symptoms have been reported in younger age groups, which are in agreement with our findings for this cohort of advanced age [38]. Studies that examined the individual components of these scores in greater detail have reported inconsistent findings regarding their specific impact, which has been attributed to differences in the study populations [38]. The relationship between ADL and OHRQoL is inconsistent in the literature. In the study that did not find such an association, conducted among disabled older individuals, the authors suggest that the overwhelming importance of other disabilities and health problems may explain the absence of this association in this particular cohort [39]. The fact that our participants do not suffer from such severe conditions may explain why we were able to observe this effect. Studies that followed the same association as in our findings identified limitations in verbal communication as a particular influence [16]. Likewise, patients’ self-rated general health status was found to influence OHRQoL, further supporting the evidence for the close link between oral health and overall health.

Our study provides novel insights by concurrently assessing oral health, OHRQoL, and geriatric parameters in community-dwelling older adults. This integrative approach goes beyond describing oral health status alone, highlighting associations and potentional intervention targets that can inform strategies to maintain oral health and quality of life in this population.

### Call for preventive strategies

After characterising oral health and examining its associations with typical geriatric syndromes and the general health status of the group, our findings underscore the broader relevance of oral health in the context of aging research. These associations, observed independently of age, emphasize the need for continued attention to the oral health of community-dwelling older people. Moreover, raising awareness of the factors influencing QoL and proactively targeting these should be considered essential goals. Community-dwelling older people are able to regularly engage with health services, as they remain relatively independent and therefore more accessible and receptive to treatment and preventive care, as exemplified by their attendance at the geriatric day clinic. However, this phase of active living is often shortened by the accumulation of comorbidities with advancing age [40]. Progressive conditions such as dementia, frailty, and probable sarcopenia are associated with limitations in daily functioning, which may be linked to reduced ability to maintain oral hygiene, attend dental appointments, or undergo treatment, potentially resulting in lower dental functional capacity [41].

### Strengths and limitations

This study has several strengths. Firstly, it draws on data from non-institutionalized community-dwelling older adults, a population that is otherwise rather difficult to access for studies. It brings more attention to an older population by providing data on a particular old age group that has been the subject of little research so far. MUSAR’s interdisciplinary collaboration applies an approach that incorporates a wide range of oral and geriatric parameters. Using validated, standardised parameters and scoring systems ensures that our results are directly comparable with findings from other research. However, certain limitations must be considered. Due to its cross-sectional design and lack of longitudinal data, the study only provides a snapshot and does not allow for causal inference or assessment of changes over time. The relatively small sample size may have limited the statistical power to detect smaller effect sizes, and missing data could not be supplemented through follow-up assessments. The lack of statistical significance for some clinical parameters in the regression analysis does not necessarily indicate the absence of an association; rather, it may reflect that our sample size was insufficient to detect these relationships. Where feasible, future research would benefit from longitudinal observation of these data and an increased sample size to enhance the robustness and generalisability of the findings. Furthermore, the generalisability of the findings is limited because the sample consisted only of older adults living in Germany, so they may not be representative of older populations in other countries.

## Conclusion

Early education, targeted prevention, and individualized treatment concepts that anticipate the aging process are crucial. Interdisciplinary collaboration could play an important role in preventing adverse outcomes, while fostering mutual benefits for the involved disciplines through shared expertise and coordinated care [17]. This may be of particular importance for our cohort of mobile, community-dwelling older adults, who remain readily accessible to medical care. Special attention should be given to transitional phases and the point of diagnosis, enabling timely interventions to limit later damage and preserve oral health and OHRQoL throughout disease progression.

## Data Availability

The data presented in this study are available on request from the corresponding author.
